# Deterioration in the Physico-Mechanical and Thermal Properties of Biopolymers Due to Reprocessing

**DOI:** 10.3390/polym11010058

**Published:** 2019-01-02

**Authors:** Jamileh Shojaeiarani, Dilpreet S. Bajwa, Chad Rehovsky, Sreekala G. Bajwa, Ghazal Vahidi

**Affiliations:** 1 Department of Mechanical Engineering, North Dakota State University, Fargo, ND 58108, USA; dilpreet.bajwa@ndsu.edu (D.S.B.); Chad.rehovsky@ndsu.edu (C.R.); ghazal.vahidi@ndsu.edu (G.V.); 2 Department of Agriculture and Biosystem Engineering, North Dakota State University, Fargo, ND 58108, USA; sreekala.bajwa@ndsu.edu

**Keywords:** recycling, thermal degradation, mechanical degradation, polylactic acid, Bioflex, Solanyl, PHBV

## Abstract

Biopolymers are an emerging class of materials being widely pursued due to their ability to degrade in short periods of time. Understanding and evaluating the recyclability of biopolymers is paramount for their sustainable and efficient use in a cost-effective manner. Recycling has proven to be an important solution, to control environmental and waste management issues. This paper presents the first recycling assessment of Solanyl, Bioflex, polylactic acid (PLA) and PHBV using a melt extrusion process. All biopolymers were subjected to five reprocessing cycles. The thermal and mechanical properties of the biopolymers were investigated by GPC, TGA, DSC, mechanical test, and DMA. The molecular weights of Bioflex and Solanyl showed no susceptible effect of the recycling process, however, a significant reduction was observed in the molecular weight of PLA and PHBV. The inherent thermo-mechanical degradation in PHBV and PLA resulted in 20% and 7% reduction in storage modulus, respectively while minimal reduction was observed in the storage modulus of Bioflex and Solanyl. As expected from the Florry-Fox equation, recycled PLA with a high reduction in molecular weight (78%) experienced 9% reduction in glass transition temperature. Bioflex and Solanyl showed 5% and 2% reduction in molecular weight and experienced only 2% reduction in glass transition temperature. These findings highlight the recyclability potential of Bioflex and Solanyl over PLA and PHBV.

## 1. Introduction

There is a huge interest in the development of bio-based materials to lessen the dependency on petroleum-based polymers owing to the growth in environmental concerns, fluctuating crude oil price, and the depletion of fossil fuels [1]. Biopolymers derived from renewable resources have a wide range of applications in different industries due to their specific characteristics. The packaging industry represents the largest demand for plastic and nearly 70% of the biopolymer global market is attributed to the packaging industry [2]. The short-term or single-use application of packaging materials is of great concern as it leads to the pollution of water. The recycling process is one of the most important alternatives to control environmental and waste management issues.

The recycling process is a waste management treatment available to lessen the environmental impact related to the disposal of plastics. Recycling not only extends the life of polymeric materials but helps the biopolymer market financially by providing circular materials of thermoplastics [3]. The recycling process involves mechanical and thermal processing [4]. In the mechanical recycling process, grinding, sorting, and drying are utilized to resize the used polymeric materials. In the thermal processing step of recycling, the used materials are subjected to high temperature. In general, the most common recycling process is melt extrusion and injection molding to reshape the used material into a new product through the application of high pressure and shear force [5].

Despite the positive aspects of the recycling process, some recycling methods result in a significant deterioration in the material properties. Loss of mechanical and thermal properties, as well as discolorations, are some common degradation issues in biopolymers as a result of the recycling process [6]. The recycled polymers should possess a set of minimum performance characters to meet the specific requirements after the recycling process. Therefore, comprehensive knowledge on the impact of successive reprocessing conditions on the physical, mechanical, and thermal properties of polymers is of paramount interest. 

This paper discusses the degradation behavior of four bio-based and bio-degradable thermoplastic polymers of major commercial importance including of poly(lactic acid) (PLA), Bioflex (PLA and co-polyester blend), Solanyl (starch-based polymer), and poly (3-hydroxybutyrate-*co*-3-hydroxyvalerate) (PHBV). Among all thermoplastic biopolymers capable of recycling, PLA is one of the most commercially available biodegradable polymers owing to its superior mechanical properties and high heat resistance [7]. Bioflex is a blend of PLA and thermoplastic copolyester (TPC). Bioflex is a biodegradable and easy processing polymer with potential applications in the food packaging and medical devices [8]. In spite of the competitive mechanical properties, to the best of our knowledge, only a small number of studies have focused on the characterization of Bioflex. Solanyl, a starch-based bio-polymer, is made out of potato starch reclaimed from potato processing [9]. Solanyl consists of oxygen-based polymers with different processing and products features. There is a low energy input to produce products from Solanyl as compared to petroleum-based polymers. PHBV with an inherent biodegradability characteristic is made by bacterial and archaea fermentation. PHBV as a member of the broad PHA (polyhydroxyalkanoates) family [1] exhibits similar mechanical properties to polyolefins. It has gained a lot of attention as an environmentally friendly substitute for petroleum-based polymers [10]. 

The goal of this work is focused on understanding the impact of processing on the properties of bio-based and biodegradable polymers. It is hypothesized that biopolymers exhibit different degradation mechanisms, as some of these polymers can be recycled without significant loss of various properties. Therefore, a comprehensive study was conducted on the influence of recycling processes on molecular weight, mechanical properties, flow characteristics, thermal properties and thermomechanical properties of PLA, Bioflex, Solanyl, and PHBV polymers after five reprocessing cycles.

## 2. Materials and Methods 

### 2.1. Materials

A list of the polymers used in this study is shown in Table 1.

### 2.2. Polymer Processing

Prior to extrusion, each polymer was dried for 24 h at 80 °C in a convection oven to remove any absorbed moisture. The polymers were extruded using a micro 18 lab-scale twin screw co-rotating extruder with a length to diameter ratio of 40/1 (Leistritz Ltd., Somerville, NJ, USA). The polymer strands were cooled in a water bath and then pelletized. The extruded pellets were then compression molded and considered as the virgin polymer (E1). The extrusion process was then repeated for four more times for each polymer to manufacture recycled polymer (E5). The temperature profile used for each polymer in the extrusion process is shown in Table 2.

### 2.3. Compression Molding

After the extrusion process, the extruded pellets were dried at 60 °C in an oven for 24 h. A compression-molding machine (Model 3856, Carver, Wabash, IN, USA) was used to manufacture test specimens. The samples were compression molded under 50 atm pressure with a platen temperature of 160 °C for 5 min. The sheets were air-dried for 5 min at room temperature of approximately 25 °C. The molded 150 mm^2^ sheets with a thickness of 4.5 mm then were cut into test specimens for various tests, following corresponding standard methods. 

### 2.4. Gel Permeation Chromatography (GPC)

Weight average molecular weight (*M_w_*), weight average molecular number (*M_n_*), and molecular weight distribution (*M_w_*/*M_n_*) of polymers were measured using gel permeation chromatography (EcoSEC HLC-8320GPC, Tosoh Bioscience, Tokyo, Japan) equipped with a refractive index detector. GPC calibration was conducted using PS standards (Agilent EasiVial PS-H 4mL, Santa Clara, CA, USA). 

Virgin and recycled polymers were dissolved in tetrahydrofuran solvent (THF) with a final concentration of 1 mg/mL. Polymer solutions were pre-filtered using a 0.22 μm syringe filter. The column temperature was 40 °C, and the solvent flow rate was set at 0.35 mL/min. Moreover, the average number of random chain scissions per unit mass of each polymer (*n_t_*) was calculated according to the following Equation (1).

(1) $$n_{t}=\left(\frac{1}{M_{nt}}\right)-\left(\frac{1}{M_{n0}}\right)$$

where, *n_t_* is the number of chain scission at a given reprocessing cycle, *M*_*n*0_ and *M_nt_* represent the number average molecular weight of samples after the initial and a given processing cycle, respectively.

### 2.5. Melt Flow Index (MFI)

Melt flow index testing was conducted in accordance with the ASTM D1238 standard. A Tinius Olsen Model MP 600 Extrusion Plastometer (Tinius Oslen Co., Horsham, PA, USA) was used to perform the testing. The applied temperatures for PLA, Bioflex, Solanyl, and PHBV were 210, 190, 170, 180 °C respectively. The applied load was 2.16 kg.

### 2.6. Differential Scanning Calorimetry (DSC)

A TA Instruments DSC Q200 (New Castle, DE, USA) was used to perform differential scanning calorimetry testing. Each sample was heated from 25 °C to 200 °C with a ramp rate of 3 °C/min. A nitrogen flow rate of 10 mL/min was used. Each sample weighed between 6 and 10 mg. The level of crystallinity of virgin (E1) and recycled polymer after five times extrusion (E5) was calculated using the following equation (Equation (2)):

(2) $$Crystallinity\;\%=\;\left(\frac{\Delta H_{m}-\Delta H_{cc}}{\Delta H_{m}^{0}}\right)\times 100$$

where ∆*H_m_* is the specific melting enthalpy of the sample, ∆*H_cc_* is the specific cold crystallization enthalpy of the sample, $\Delta H_{m}^{0}$ is the melting enthalpy of the 100% crystalline polymer. The melting enthalpy of 100% crystalline PLA was 93.7 J/g. Since Bioflex is a PLA-based polymer, 93.7 J/g was also used as its melting enthalpy. The melting enthalpy of 100% crystalline PHBV was 146 J/g [2].

### 2.7. Dynamic Mechanical Analyzer (DMA)

A TA Instruments DMA Q800 (New Castle, DE, USA) was used to evaluate the dynamic mechanical behavior of each polymer. Measurements were conducted in a dual cantilever mode and the temperature swept from 25 to 90 °C at a heating rate of 1 °C/min, with a fixed frequency of 1 Hz. A preload force of 0.015 N was applied, and the oscillation amplitude was 15 μm for PLA, Bioflex, and PHBV. An amplitude of 5 μm was used for Solanyl to prevent yielding during the test. The sample dimensions were 60 mm × 4.5 mm × 1.5 mm, and the initial length of the specimen was measured using the dynamic mechanical analyzer once it was secured in the clamp. The storage modulus and damping coefficient (tanδ) were recorded as a function of temperature. 

### 2.8. Thermogravimetric Analysis (TGA)

Thermogravimetric analysis of polymers was performed using a Perkin Elmer model (Shelton, CT, USA) analyzer in a nitrogen atmosphere (10 mg/min). The heating rate was 10 °C/min, and the determination range was 25–600 °C. The weight lost and the first derivative data (DTG) of TGA were obtained to calculate the thermal degradation behavior in polymers. *T*_onset_ (the temperature at which the polymer starts to degrade), *T*_peak_ (the temperature corresponding to the maximum degradation rate), and *T*_endset_ (final temperature at which the degradation process ends) were reported for virgin and recycled biopolymers.

### 2.9. Flexural Testing

The Three-Point bending test was carried out to determine the flexural strength on standard beam specimens of size 150 mm × 13 mm × 4.5 mm and support span of 75 mm. The test was conducted using a universal testing machine, Instron Model 5567 (Norwood, MA, USA) following ASTM D790 standard. The Instron machine was equipped with a load cell of 2 kN and the crosshead speed was set at 2 mm/min. 

### 2.10. Izod Impact Testing

Izod impact testing was conducted in accordance with the ASTM D256 standard using Tinius Olsen impact tester (IT504, Tinius Olsen, Horsham, PA, USA). The pendulum weight and radius were 4.497 kg and 334.949 mm, respectively. No additional load was added to the pendulum for PLA, Solanyl, and PHBV. Weights of 0.453 kg were assigned to each side of the pendulum for Bioflex to achieve complete breaks. Test sample dimensions were 63.5 mm × 13 mm × 4.5 mm with a 2 mm notch in the middle of the sample. 

### 2.11. Statistical Analysis 

Minitab software version 17 (Minitab Inc., State College, PA, USA) was used to statistically analyze the mean values and standard deviations of the mechanical properties (Flexural testing, Izod impact testing, and DMA). The analysis of variance (ANOVA) and Tukey’s test with a significance level of 5% (α = 0.05) was used to analyze the data. Eight replicates of each formulation were tested in Flexural testing and Izod impact testing. In the case of DMA, for each formulation, three replicates were used, and the mean values are reported.

## 3. Results

### 3.1. Gel Permeation Chromatography (GPC)

In order to investigate the influence of the successive recycling process on the molecular structure of the polymers, GPC analysis was performed and the weight average molecular weight (*M_w_*), the number average of molecular weight (*M_n_*), and the molecular weight distribution (*M_w_*/*M_n_*) are all reported in Table 3. It can be seen that for all polymers with a thermo-mechanical history of the successive recycling process, *M_w_* and *M_n_* values decreased. These observations indicate that chain scission occurs especially in long chains, as a result of high shear and high temperature during the extrusion process. Scaffaro et al. reported similar observations in PLA with increasing number of recycling processes [3]. The higher decline in *M_w_* was observed in PHBV by 82% reduction, followed by PLA by 78%, suggesting the structure of PHBV and PLA are more susceptible to high shear and temperature in comparison with Bioflex and Solanyl. Indeed, chain scission mainly occurs in polymers with higher *M_w_* (PHBV and PLA) to form polymer chains of medium size. However, Bioflex and Solanyl with relatively lower *M_w_* as compared to PLA and PHBV exhibited a negligible reduction in *M_w_* (5% and 2%) after five reprocessing cycles. PLA and PHBV experienced a noticeable reduction in the molecular weight distribution (*M_w_*/*M_n_*) by about 15% and 31%, respectively (see Table 3), indicating that recycled PLA and PHBV have a more homogenous composition than virgin polymers. An increase in molecular weight distribution indicates that recycled Bioflex and Solanyl are more polydisperse than the corresponding virgin polymers. Martin et al. reported that polymeric materials consisting of a blend of two or more polymers possess a wider range of molecular weight after the recycling process [4]. 

As expected, one can observe that PLA and PHBV exhibited higher values for the average number of random chain scission per unit mass (*n_t_*) after five times of reprocessing cycles (Table 3). In fact, higher values for *n_t_* indicates that the polymer chains are more sensitive to thermomechanical degradation. On the contrary, the lower values for *n_t_* in Bioflex and Solanyl can be probably attributed to the lower rate of polymer chain scissoring due to the cross-linking phenomenon which occurs in polymers above the melting temperature [5]. Moreover, the most probable reaction involved in the thermal degradation of polymers with a considerable reduction in molecular weight is random scission occurring as a result of high temperature and high shear force. 

**Figure 1:**
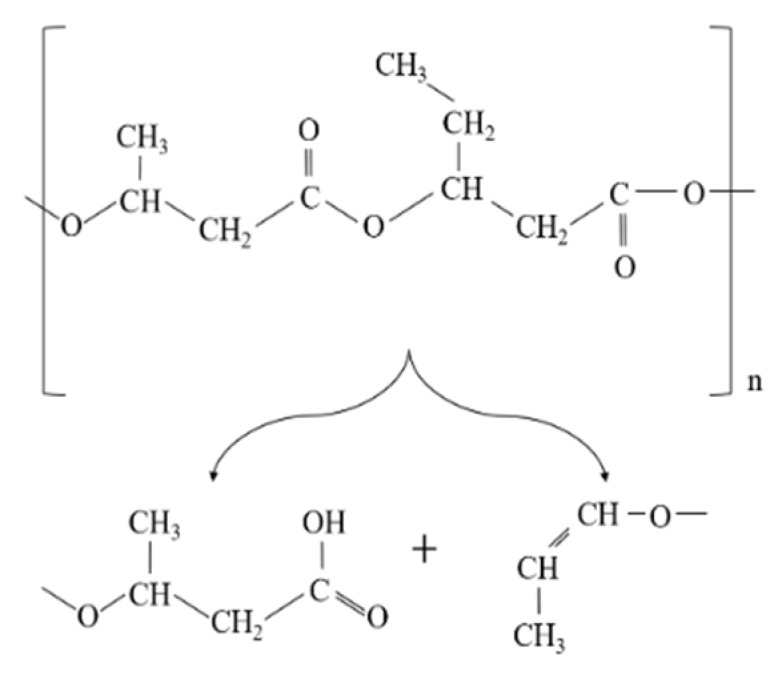
Schematic diagram of poly (3-hydroxybutyrate-*co*-3-hydroxyvalerate) (PHBV) random chain scission during thermal degradation.

In PHBV polymer, the significant reduction in molecular weight of the polymer can be attributed to the unzipping reaction in PHBV through cis-elimination mechanism (Mclafferty arrangement) just above its melting temperature [6]. The formation of a variety of oligomers after degradation is illustrated in Figure 1 [7]. 

**Figure 2:**
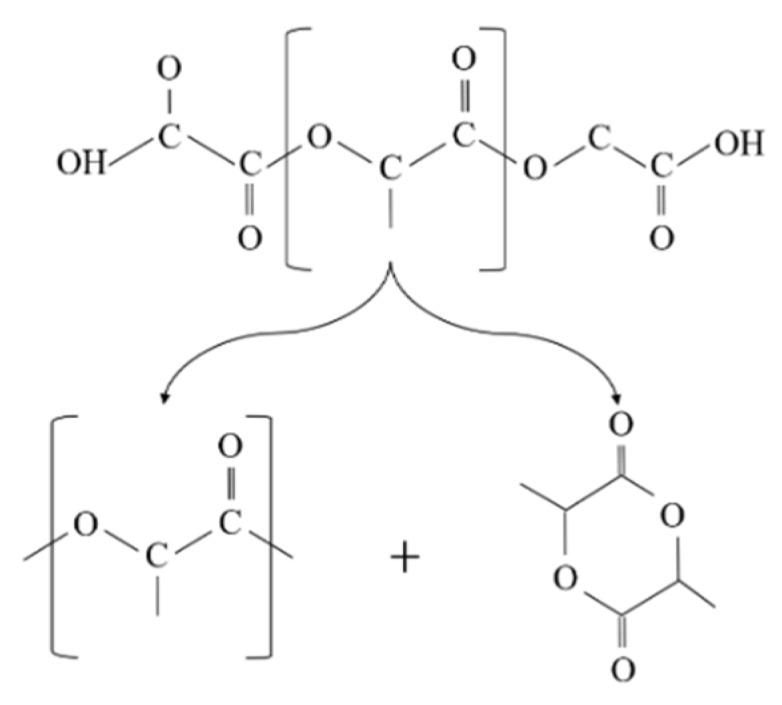
Schematic diagram of poly(lactic acid) (PLA) random chain scission during thermal degradation.

Similarly, the degradation of PLA is because the generation of acidic molecules act as catalyst to accelerate the degradation. The considerable reduction in the molecular weight of PLA was more likely associated with the thermal degradation caused by polymer chain scissions into linear and cyclic oligomers [8] (Figure 2). 

In addition, changes in the molecular weight of PLA can be linked to the cleavage of the long chains to shorter ones due to hydrolysis and intermolecular transesterification. The small value of the activation energy for the thermal degradation of PLA (21–23 kJ/mol) in comparison to other polymers indicates that PLA is highly sensitive to thermal treatment [9]. 

**Figure 3:**
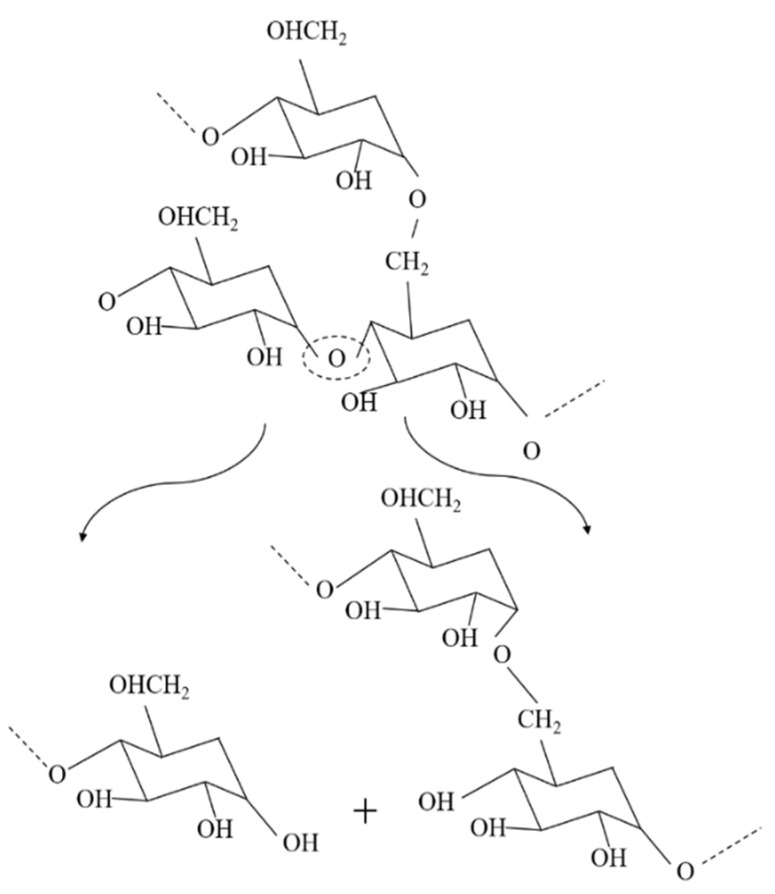
Schematic diagram of Solanyl thermal degradation and molecular cleavage.

The molecular weight of the recycled Solanyl exhibited a limited reduction by 2% after five times of the recycling process. This observation can probably be attributed to either a less sensitive structure to the recycling process or the crosslinking phenomenon which occurs in recycled polymers as a result of subsequent recombination in the molecular chain cleavage as evidenced by Kale et al. They claimed the formation of new crosslinking in the structure of polymers under high temperature [10]. Solanyl is a starch-based polymer, having a similar chemical structure to starch; the probable thermal decomposition for Solanyl could be as illustrated in Figure 3. The starch-based polymers have a complex thermal degradation [11] and the chemical structure of the polymer ruptures to release the constituent molecules.

The complex nature of starch contains amylose and amylopectin homopolymer blends. Starch is reported to undergo thermal reactions around 300 °C [11] anda lower processing temperature was used in this study (Table 2). It has been reported that degradation of these components leads to the formation of interpenetrating structures or helical structures which crystallize. Generally, starch thermal degradation leads to the formation of hydroxyl, ether links, as well as de-hydration of hydroxyl groups present on the glucose ring which we did not observe in FTIR results (not included in this study). 

In Bioflex, which is a PLA-starch blend, 5% reduction in the *M_w_* was observed. To improve the compatibility, interfacial adhesion, and enhance the dispersion between the two polymers, glycerol, formamide, and water are used as plasticizers. In the presence of heat and moisture, the strong intermolecular and intramolecular hydrogen bonding between polymers and plasticizers weakens which results in degradation of polymer. In Bioflex, degradation primarily occurs through the chain scission reaction of PLA. Bioflex is a USDA certified bio-based product. It is a polymer blend with PLA as the main component and it is certified as compostable material according to European standard (EN 13432). There is no information regarding its chemical structure, however, the molecular weight, flow rate, mechanical properties, and thermal properties of this polymer are reported in this work.

### 3.2. Effect of Recycling on Flow Characteristics

The flow characteristics of polymer melts were studied, and Table 4 displays the melt flow index of virgin and recycled polymers. The melt flow index increased by 71% for PLA, 44% for Bioflex, 13% for Solanyl, and 108% for PHBV as the number of the extrusion process increased from one to five. Collins and Metzger reported a relationship between flow activation energy and molecular weight of polymers. They found that flow activation energy in polymers is inversely proportional to the molecular weight [12]. Therefore, the observed increment in the melt flow index was a result of the reduction in the molecular weights (Table 3). The inverse relationship between melt flow index and molecular weight in polymers can explain the significantly higher increase in the melt flow index in recycled PLA and PHBV as compared with their corresponding virgin polymers.

### 3.3. Differential Scanning Calorimetry (DSC)

**Figure 4:**
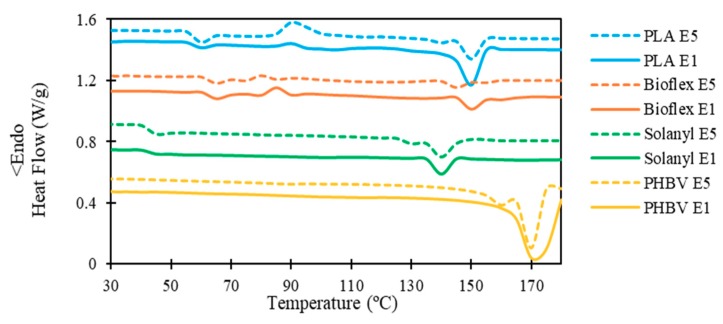
Representative DSC analysis curve of virgin and recycled polymers.

The influence of the recycling process on the thermal properties and degree of crystallinity of the polymers is shown in the DSC heating thermograms in Figure 4 and the data are summarized in Table 5. 

#### 3.3.1. PLA

From the thermographs, virgin PLA showed a slightly higher glass transition temperature (*T_g_*) in comparison with its recycled one. The Flory-Fox equation (Equation (3)) can be employed to explain the observation in *T_g_* of the polymers regarding the molecular number:

(3) $$T_{g}=T_{g\infty }-\frac{K}{M_{n}}$$

In which, *T_g_* is the glass transition and *T_g∞_* reveals the maximum glass transition temperature which occurs theoretically for the polymer with an infinite molecular weight. *K* is an empirical constant associated with the free volume in the polymer chains. In fact, polymer samples with lower molecular number possess more free volume associated with the end group in the polymer chains, inducing chain mobility. This, in turn, can facilitate the transition from a glassy to a rubbery state and thereby reducing the glass transition temperature [13]. 

No significant change was observed in the crystallization temperature, and the recycling process hardly changed the crystallization temperature of PLA. However, PLA E5 possessed a higher exothermic peak at the crystallization point (i.e., (∆H_c_)E1 < (∆H_c_)E5). This observation confirmed that less energy is required for the crystallization process in PLA E1, and these results let us propose that the degree of crystallinity of PLA E1 could be higher than PLA E5. This hypothesis was confirmed by calculating the degree of crystallinity using Equation 1 (Table 5). The substantial decrease in the degree of crystallinity in PLA E5 as compared with PLA E1 was probably attributed to the radical reaction as a result of the thermal degradation of PLA during the recycling process [14]. 

**Figure 5:**
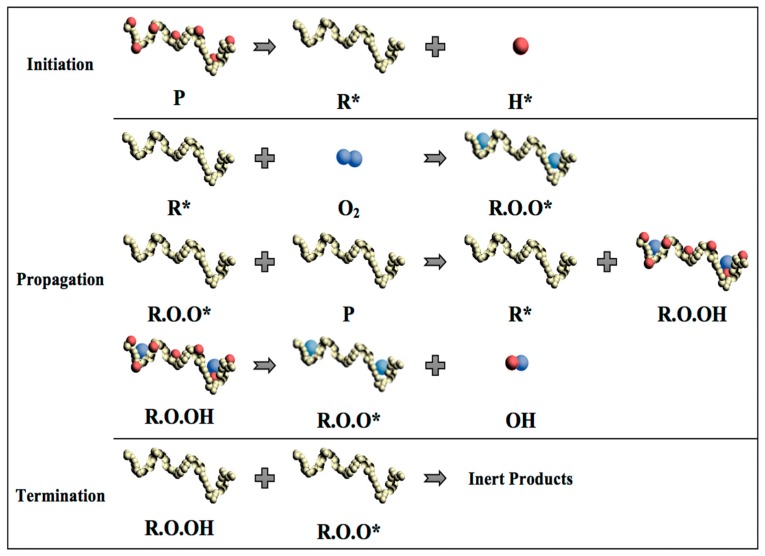
Schematic diagram of polymer chain thermal degradation and formation of free radicals. (P: polymer chain; R*: Free radical; H*: Hydrogen ion; O_2_: Oxygen molecule; R.O.O*: proxy radical; R.O.OH: Hydroproxide; RO* and *OH: Free radical).

In general, the thermal degradation of polymers includes three steps as initiation, propagation, and termination [15]. In the initiation step, the loss of a hydrogen atom from the polymer chain occurs when heat is imposed on the polymer chain as the energy input. This process leads to the formation of highly reactive and unstable free radical (R*) and a hydrogen ion with an unpaired electron (H*). In the propagation step, the free radical reacts with an oxygen molecule (O_2_), forming a proxy radical (ROO*) with a high level of activity and capable of removing a hydrogen atom from another polymer chain to form a hydroperoxide (ROOH). Therefore, the free radical regeneration occurs in the propagation step. Moreover, the hydroperoxide splits into two new free radicals, (RO*) and (*OH), which will propagate the chain degradation to form other polymer molecules. The final step of polymer chain degradation (termination) is attained through ‘mopping up’ the free radicals to form inert products. This step takes place naturally by combining free radicals or it can be aided by using stabilizers in the plastic (Figure 5).

A single melting peak in the range of 150–152 °C was observed in PLA E1, however, PLA E5 exhibited a bimodal endothermic peak owing to the formation of different crystalline structures with different sizes and degree of perfection during successive recycling processes [16]. 

#### 3.3.2. Bioflex

For Bioflex, the glass transition temperature experienced a non-significant decrease as the number of recycling process increased. This observation indicates a moderate shortening of the polymer chains after recycling, which means a slightly higher segmental chain mobility in Bioflex E5 as compared to E1. Morreale et al. reported a similar observation for Bioflex-based composites as a result of reprocessing [17]. Chain scission and crosslinking occurs simultaneously, and chain scission in polymer chains lowers the crosslink density, and results in the formation of more or longer dangling chain ends [18].

Similarly, the crystallization temperature exhibited a non-significant decrease after five reprocessing cycles, suggesting that crystallization took place at a lower temperature for Bioflex E5. This observation proves that polymer chains scissor after recycling processing which facilitates the crystallization kinetics [19]. No significant change was observed in the melting points of Bioflex. A slightly lower degree of crystallinity was observed in Bioflex E5 as compared to Bioflex E1 (1.8%). The reduction in the level of crystallinity can be attributed to the crosslinking phenomenon, which occurred in the polymer during exposure to either heat or shear above its melting point. In general, the degree of crystallinity in the polymer decreases with an increase in the crosslinking [20]. This observation can provide further proof of the cross-linking phenomenon in Bioflex, which limited the decline in *M_w_* of Bioflex after the recycling process (see Table 3). After polymer chain branching, some of the polymer chains become linked together which results in cross-linking and embrittlement of the polymer.

#### 3.3.3. Solanyl

As expected from the Florry-Fox equation, no significant decrease was observed in the glass transition temperature of Solanyl as the number of recycling processes increased. Moreover, cross-linking formation between the polymer chains above their melting point which was confined to the polymer chains and hindered the transition from glassy rigid to a rubbery state, was compensated by the scissoring process in Solanyl E5. Therefore only a non-significant decrease was observed in the glass transition temperature of Solanyl E5 in comparison with Solanyl E1. Crystallization was not likely to have occurred since no crystallization peak was observed in virgin and recycled Solanyl. This could be attributed to the slow crystallization in Solanyl. In addition, the shortness of the side-chain length in Solanyl might be another reason for the poor crystallization ability in Solanyl. A bimodal melting peak was observed in Solanyl E5 with a big peak at 140.53 °C and a smaller peak at 130.34 °C, while Solanyl E1 exhibited one endothermic peak at a slightly higher temperature (141.22 °C). The bimodal endothermic peaks in Solanyl E5 might be attributed to the presence of a species with a lower molecular weight (*M_w_*) than the original material formed as a result of chain scission caused by different resistance to heat [21].

#### 3.3.4. PHBV

Neither the glass transition nor the crystallization temperature was observed in PHBV polymer, which was attributed to either the slow crystallization rate in PHBV [22] or the occurrence of crystallization out of the range of the temperature studied in this work [23]. PHBV E5 exhibits two melting peaks; a large peak at low temperature (165.25 °C) and a small peak centered at high temperature (175.11 °C). This bimodal melting peak occurred because the imperfect crystals had adequate time to melt and reorganize into crystals with higher structural perfection. However, PHBV E1 melting possessed only one peak. No significant change was observed in the degree of crystallinity.

### 3.4. Dynamic Mechanical Analyzer (DMA) 

**Figure 6:**
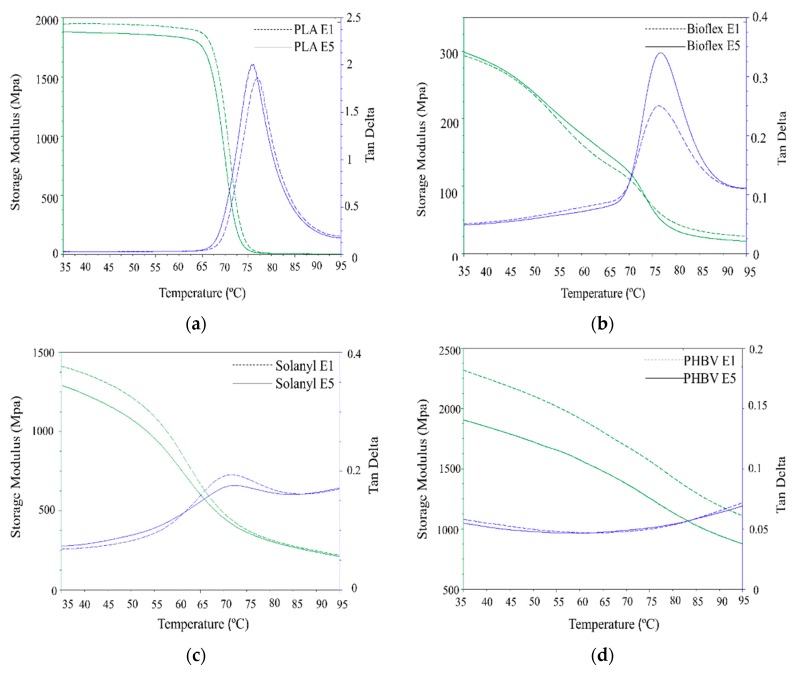
DMA analysis curve of virgin (E1) and recycled (E5) polymers: (**a**) PLA; (**b**) Bioflex; (**c**) Solanyl; and (**d**) PHBV.

The DMA investigation was performed to study the influence of reprocessing cycles on the dynamic-mechanical performance of the polymers. Figure 6 illustrates the storage modulus (E’) and the mechanical loss factor (tanδ) for virgin (E1) and recycled (E5) polymers as a function of temperature. All the polymers experienced a declining trend in storage modulus throughout the whole temperature range used in this study. In the case of PLA, a drastic drop was observed in the vicinity of the glass transition temperature. This behavior can be attributed to an increase in the molecular mobility of the polymer chains above the *T_g_*. However, Solanyl, Bioflex, and PHBV experienced a gentle decrease throughout the whole temperature range.

#### 3.4.1. PLA

PLA exhibited a non-significant decline in storage modulus in the glassy (7.25%) and leathery regions with an increase in the number of reprocessing times. The lower storage modulus in recycled PLA can be attributed to the lower molecular weight in the recycled polymers caused by thermomechanical degradation occurring during the reprocessing polymer degradation [24]. tanδ, as the mechanical loss factor, indicates the energy dissipation of materials under cyclic load at elevated temperatures. A higher tanδ intensity indicates more viscous (dashpot-like) behavior than elastic (spring-like) nature in materials [25]. It is seen from Figure 6a that, the recycled PLA exhibited significantly higher tanδ peak intensity (6.6%) as compared to virgin PLA, suggesting more viscoelastic behavior in recycled PLA than virgin PLA. This observation can be explained by the fact that the decrease in *M_w_* of the polymer can increase segmental motion, hence, leading to more viscous behavior in the polymer. A similar observation was reported for different polymers as a result of the recycling process [26]. 

#### 3.4.2. Bioflex

Storage modulus curves were almost identical for both virgin and recycled Bioflex, and no significant change was observed in the glassy state. However, Bioflex E5 exhibited a slightly higher storage modulus in the leathery region (Figure 6b) and in the rubbery state Bioflex E1 was somewhat stiffer than virgin Bioflex. This was mainly ascribed to the efficient mechanical interlocked cross-linking of the polymer chain, which partially strengthen the polymer at a temperature above the glass transition temperature in the rubbery state. Similar behavior was reported for Bioflex reinforced with wood fibers [17]. In the tanδ curve, the peak intensity becomes significantly higher (45%) upon recycling, indicating a significant change in the molecular structure of recycled Bioflex as supported by the molecular weight values. The lower molecular weight makes the segmental motion easier and this, in turn, can increase the viscoelastic properties (damping behavior) in polymers [27].

#### 3.4.3. Solanyl

Solanyl E5 exhibited a non-significantly lower storage modulus in the glassy (4.2%) and rubbery regions as compared with Solanyl E1, however, this discrepancy decreased as the temperature increased (Figure 6c). This result can be an indicator of the limited segmental mobility in the polymer chain due to the presence of the cross-links in the recycled Solanyl network. The formation of cross-linking in the polymer matrix hinders the untangling of the polymer chains and the sliding over each other after the glass transition temperature in the rubbery state [28]. In the DMA test, the mobility of the polymer chains is a response against the dynamic deformations of a particular frequency, and the viscoelastic properties of the polymers can be evaluated through the peak values of the tanδ curves [29]. Surprisingly, the recycled Solanyl displayed a lower tanδ peak intensity as compared with virgin Solanyl, suggesting, fewer polymer chains participate in the transition from a glassy to a rubbery state. 

#### 3.4.4. PHBV

A significant decrease occurred in the storage modulus of recycled PHBV throughout the temperature range studied in this work, because of thermomechanical degradation during the reprocessing cycles (see Table 3). In fact, an 80% decline in *M_w_* led to a decrease of 20% in storage modulus of PHBV E5 in comparison with PHBV E1. In the case of tanδ, a completely different behavior was observed for PHBV, and the tanδ was almost temperature-independent, indicating that extrusion temperature had little or no effect on the viscosity of the polymer in the temperature range studied in this work.

### 3.5. Thermogravimetric Analysis (TGA) 

Thermogravimetric analysis (TGA) and the derivative thermogravimetry (DTG) curve were used for assessment of the thermal stability of virgin and recycled polymers. The area of the DTG peak is directly proportional to the mass loss of the polymer over the same temperature range and the DTG peak height indicates the rate of mass loss at the corresponding temperature.

#### 3.5.1. PLA

**Figure 7:**
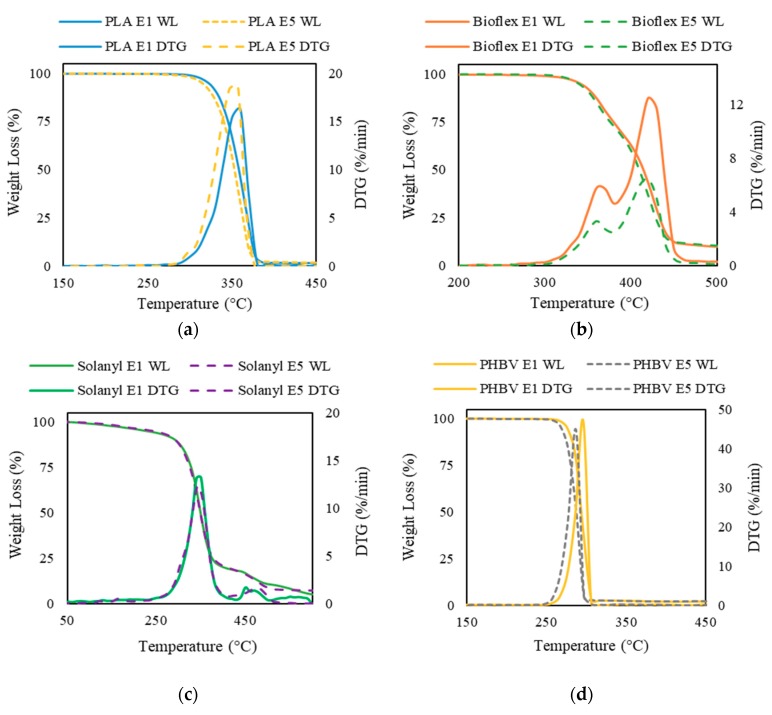
Thermogravimetric traces of virgin polymers (solid line) and recycled polymers (dashed line): (**a**) PLA; (**b**) Bioflex; (**c**) Solanyl; and (**d**) PHBV.

Figure 7a exhibits that the weight-loss trend for virgin and recycled PLA is almost identical. A single step decomposition in a narrow temperature interval was observed for PLA E1 and PLA E5. The mass started decreasing at temperatures of 287 °C and 294 °C in recycled and virgin PLA, respectively. Increase in the number of reprocessing cycles led to a slight decrease in the *T*_onset_. Typically, an increase in the *T*_onset_ illustrates a more thermally stable polymer [30]. The observation that virgin PLA was more thermally stable as compared with recycled PLA was probably attributed to the molecular weight reduction that took place during the recycling process. It is reported that there is a linear relationship between thermal stability (referred as *T*_onset_) and the average molecular weight, in which, the higher molecular weight can result in more thermally stable polymer materials [31,32]. As expected from the single step weight loss in TGA curve, only one peak was noticed in the DTG curve. The *T*_peak_ shifted to a lower temperature for recycled PLA. Moreover, the DTG peak value was higher for the recycled polymer, suggesting a higher thermal degradation rate in PLA E5 as compared to PLA E1. These observations occurred because less energy is needed to degrade polymers with lower molecular weight. 

#### 3.5.2. Bioflex

The thermogram of Bioflex E1 exhibits two degradation steps and the second step dominates the overall weight loss (Figure 7b). In the first stage, a weight loss of about 18 wt % occurred between ~300–380 °C; whereas a further 81 wt % loss was verified between ~380–470 °C during the second degradation step. The maximum rate of weight loss was centered at ~420 °C. A similar degradation profile but lower loss rate was observed in the thermal decomposition of Bioflex E5. The first degradation step in the temperature range of ~300–370 °C contributed towards the 32 wt % of the weight loss and the main weight loss (by 66 wt %) occurred in the temperature range of 385–450 °C. *T*_onset_ in recycled Bioflex shifted slightly to lower temperature as compared to virgin Bioflex by about 2 °C. This result implies that a higher activation energy is needed to decompose Bioflex E1, which can be explained by the higher molecular weight of Bioflex E1. The solid residue of the Bioflex E1 and E5 was 8.7 wt %, and 7.8 wt %, respectively. This indicates an incomplete thermal decomposition of both Bioflex E1 and E5.

#### 3.5.3. Solanyl

No notable differences were observed in TGA results for Solanyl E1 and E5 up to 372 °C (Figure 7c). Both virgin and recycled Solanyl decomposed within a relatively wide temperature interval through a multi-step decomposition. The initial weight loss occurred as the temperature increased from room temperature to ~230–240 °C. During this stage, a weight loss of 6 wt % and 10 wt % were observed for virgin and recycled Solanyl, indicating moisture evaporation and dehydration. The main weight loss took place in the range of 250–425 °C. This step was accompanied by 80% of weight loss recorded on the TGA curve. The higher loss rate in Solanyl E1, indicates faster decomposition in Solanyl E1 than Solanyl E5. The solid residue left from Solanyl E1 at 600 °C was slightly higher than Solanyl E5 and resembles a foam structure, indicating the formation of oxidation products during heating. It was reported that thermal decomposition at a relatively high rate could make a close pack, forming a solid shell layer which inhibits the degradation of the inner layers and increases the char residue in carbohydrate polymers [33].

#### 3.5.4. PHBV

The TGA curves of virgin and recycled PHBV are illustrated in Figure 7d. A single step weight loss in a narrow temperature range of 251–297 °C and 256–306 °C was inspected for virgin and recycled PHBV, respectively. The *T*_onset_, *T*_peak_, and *T*_endset_ shifted to a lower temperature for recycled PHBV. Hence, it can be concluded that virgin PHBV needs more activation energy for decomposition and degradation.

### 3.6. Effect of Recycling on Mechanical Properties 

The mechanical properties of virgin and recycled polymers were studied through flexural and impact tests and the results are summarized in Table 6. PLA, Bioflex, and PHBV exhibited a slight reduction of 3.5%, 8.7%, and 1.7% in flexural strength on increasing the number of reprocessing cycles. These observations can probably be attributed to the lower molecular weight in the recycled polymers because of the deterioration of the polymer chains during successive extrusion cycles. In general, the mechanical properties of biopolymers are strongly dependent on *M_w_*. Therefore, any changes in the polymer chain as a result of degradation due to high temperature and shear in the extrusion process are likely to influence the mechanical properties. 

Flexural strength was significantly lower for recycled PLA and PHBV as compared to their corresponding virgin counterparts, however, no significant change was observed in Bioflex and Solanyl after the reprocessing cycle, which is more likely due to the reduction trend in the molecular weight in the recycled polymers (see Table 3). 

The flexural modulus for all polymers shows a decreasing trend as the number of recycling processes was increased. A higher decline in flexural modulus was observed in PHBV E5 as compared to PHBV E1 by 11% followed by PLA by 9% decrease. These observations can be attributed to the fact that the recycling procedure includes polymer crushing and melting, which leads to polymer chain degradation. In addition, thermal degradation in the melting process contributes to the failing of intermolecular bonding between the polymer chains and the flexural strength in polymers tends to decrease as the number of recycling processes increased [34,35]. 

The impact strength of the polymers is shown in Table 6. It can be seen that all recycled polymers exhibit lower impact strength as compared with their corresponding virgin polymer. These results are consistent with another work which reported lower impact strength for PBT/PC/ABS after five times of the reprocessing cycle [36]. This may be explained by the lower molecular weight of recycled polymers due to the degradation phenomenon caused by heating and shear stress through the recycling process. PHBV with the higher reduction in molecular weight (Table 3) showed more reduction in impact strength after five times of reprocessing cycles (30%). Although Solanyl exhibited lower impact strength as compared with other polymers, the results showed that the effect of the recycling process on the impact strength was not significant and the impact strength remained constant after the recycling process.

## 4. Conclusions

In this study, detailed experimental analyses of molecular structure, the mechanical and thermal properties of recycled PLA, Bioflex, Solanyl, and PHBV after five extrusion and molding cycles were carried out. The molecular weight of the aforementioned polymers was studied using GPC. The mechanical properties were tested through flexural and impact tests. DSC was used to investigate the thermal properties of the virgin and recycled polymers and the overall conclusions can be summarized as follows:

1. PHBV and PLA exhibited a considerable reduction in molecular weight after five times of undergoing extrusion cycles.
2. DMA results showed a higher storage modulus for virgin polymers and a more viscoelastic behavior in the recycled polymers.
3. Solanyl exhibited better thermal processability compared to the other biopolymers studied in this work. The molecular structure of Solanyl possessed lower recycling sensitivity.
4. The maximum flexural strength in all polymers except Solanyl declined as the number of extrusion cycles increased.

## Figures and Tables

**Table 1:** List of Polymers used in this study.

Polymer	Grade	*M_w_* (g/mol) *	Supplier
PLA	2003D	200,000	NatureWorks LLC (Minnetonka, MN, USA)
Bioflex	F-2110	53,000	FKuR Kunststoff GmbH (Willich, Germany)
Solanyl	C2201	NA	Rodenburg Biopolymers (Oosterhout, Netherlands)
PHBV	Y1000P	300,000	TianAn Biopolymer (Ningbo, China)

* The specifications are derived from the supplier technical bulletin.

**Table 2:** Extrusion temperature profiles for each polymer.

Extruder Temperature at Different Zones (°C)										
Polymer	Zone 1	Zone 2	Zone 3	Zone 4	Zone 5	Zone 6	Zone 7	Gate Adaptor	Die	Screw RPM
PLA	152	154	157	160	160	163	166	168	160	180
Bioflex	154	160	166	171	177	179	182	185	170	200
Solanyl	93	121	132	135	138	141	143	146	140	120
PHBV	177	166	160	154	149	143	138	132	125	200

**Table 3:** The number averaged (*M_n_*), weight averaged molecular weight (*M_w_*), disparity, and average number of random chain scissions (*n_t_*) of biopolymers.

Polymer	Extrusion Cycles	Molecular Weight (g/mol)	Molecular Number (g/mol)	(*M_w_*/*M_n_*)	*n_t_*
PLA	1	203,500	152,245	1.3	1.9 × 10^−5^
	5	44,149	39,663	1.1	
Bioflex	1	52,132	39,759	1.3	7.2 × 10^−6^
	5	49,276	30,891	1.5	
Solanyl	1	61,109	31,047	1.9	2.4 × 10^−6^
	5	59,695	28,885	2.0	
PHBV	1	298,500	176,383	1.6	1.5 × 10^−5^
	5	52,262	48,525	1.1	

**Table 4:** Melt flow index of virgin and recycled biopolymers.

Polymer	Extrusion Cycles	Melt Flow Index (g/10 min)
PLA	1	10.60
	5	18.20
Bioflex	1	6.37
	5	9.18
Solanyl	1	12.73
	5	14.51
PHBV	1	18.18
	5	37.90

**Table 5:** Evaluation of the thermal properties of polymers as a function of the recycling process.

Polymer	Extrusion Cycles	*T_g_* (°C)	*T_c_* (°C)	*T_m_* (°C)	*X*%
PLA	1	60.36	90.87	150.86	33.17
	5	55.04	91.23	151.87	7.01
Bioflex	1	65.73	85.34	151.36	2.72
	5	64.23	80.24	148.12	2.67
Solanyl	1	45.23	-	141.22	-
	5	44.40	-	140.53	-
PHBV	1	-	-	168.57	55.91%
	5	-	-	175.62	54.46%

*T_g_*: Glass transition temperature; *T_c_*: Crystallization temperature; *T_m_*: Melting temperature; *X*%: Degree of crystallinity.

**Table 6:** Changes in mechanical properties of the polymers after the recycling process.

Polymer	Extrusion Cycles	Flexural Strength (MPa)	Flexural Modulus (GPa)	Impact Strength (kJ/m^2^)
PLA	1	77.7 ± 7.4 ^a^	2.5 ± 0.7 ^a^	7.7 ± 0.2 ^a^
	5	75.0 ± 3.4 ^b^	2.2 ± 0.1 ^b^	7.0 ± 0.1 ^b^
Bioflex	1	9.0 ± 0.8 ^a^	0.3 ± 0.0 ^a^	9.1 ± 0.5 ^a^
	5	8.2 ± 0.2 ^b^	0.2 ± 0.0 ^b^	8.7 ± 0.2 ^b^
Solanyl	1	15.1 ± 0.4 ^a^	1.4 ± 0.0 ^a^	2.1 ± 0.4 ^a^
	5	15.2 ± 0.1 ^a^	1.3 ± 0.1 ^b^	2.1 ± 0.3 ^a^
PHBV	1	47.4 ± 3.6 ^a^	3.2 ± 0.3 ^a^	3.7 ± 0.1 ^a^
	5	46.5 ± 4.9 ^b^	2.7 ± 0.2 ^b^	2.6 ± 0.1 ^b^

Different letter superscripts for each of the polymers’ properties indicate a statistically significant difference (*p* < 0.005).
